# Ultrasonic Inspection of Localized Defects in Low-Porosity CFRP

**DOI:** 10.3390/s19071654

**Published:** 2019-04-06

**Authors:** Wei Feng, Xiaojun Zhou, Xiang Zeng, Chenlong Yang

**Affiliations:** 1 State Key Lab of Fluid Power and Mechatronic Systems, Zhejiang University, Hangzhou 310027, China; fengweizju@126.com (W.F.); cmeesky@163.com (X.Z.); 2 CRRC Zhuzhou Institute Co. Ltd., Zhuzhou 412001, China; zzjjuu0104@163.com

**Keywords:** CFRP laminate, backscattered signal model, localized defects, variational mode decomposition

## Abstract

A preliminary backscattered signal model of carbon-fiber-reinforced plastic (CFRP) laminate was established. The backscattered signal model was composed of three sub models, which were concerned with structural signal, scattering signal, and non-acoustic noise. Resonance in structural signal and echoes excited by defects (porosity and rich-resin) were studied. The results showed that: resonance would occur when there was sufficient bandwidth; when the CFRP laminate contained voids, the center frequency of the backscattered signal decreased; and the localized defects, including rich-resin and localized porosity, tended to generate apparent echoes where they located. A simplified backscattered signal model was subsequently put forward, showing certain potential in revealing time-frequency properties of backscattered signals. The newly proposed variational mode decomposition was used for defect modes extraction, successfully avoiding the mode mixing and false modes which easily exist in empirical mode decomposition. Subsequently, the generalized Stockwell transform was adopted for the defects localization. The simulation and experiment denoted the coincidence between the backscattered signal model and the experimental signal, and showed the effectiveness of variational mode decomposition and generalized Stockwell transform in localized defects detection.

## 1. Introduction

Carbon-fiber-reinforced plastic (CFRP) has been widely used in the aeronautical industry due to its low density and high strength. However, micro-defects including void, rich-resin, crack, delamination, etc., usually lead to performance degradation of CFRP workpieces. With the recent rapid growth of demand for CFRP, ultrasonic techniques have been used for non-destructive extensive testing, and remarkable progress has been made. In addition to traditional methods such as the ultrasonic attenuation method, the ultrasonic velocity method and the acoustic impedance method [1,2,3,4], methods based on analysis of backscattered signals have received great attention since they still work in cases without back-wall echo [5,6,7,8]; however, current works have mainly concentrated on modelling the relationships between CFRP porosity and certain features (amplitude, energy, etc.) of the backscattered signal by means of experimental testing. Apart from those experimental methods, numerical methods [9,10,11,12,13] have also been widely used for acoustic characterization.

In this article, a preliminary backscattered signal model of CFRP laminate was established and verified by experiment. Sub-models for the structural signal, scattering signal, and non-acoustic noise were established, subsequently forming the backscattered signal model. Based on study of time-frequency characteristics of the sub-models, a simplified backscattered signal model was put forward. Finally, the variational mode decomposition (VMD) [14] was implemented for defect modes extraction, and the generalized Stockwell transform (GST) [15,16] was adopted, achieving localized defects detection in the laminate.

## 2. Backscattered Signal Modelling

When the incident ultrasonic wave propagates into the CFRP laminate, the backscattered signal will be mainly composed of three components: (1) the structural signal due to multiple reflections and interference for the multi-layered structure; (2) the scattering signal due to the porosity and rich-resin; and (3) the non-acoustic noise due to the measurement instruments and environment. Here, the laminate is assumed as having no defects with a large size (delamination, for instance) since it is easy to identify the clear flaw echo in the backscattered signal. Scattering signals excited by the carbon-fiber are negligible, as they are too weak compared with those due to the porosity.

### 2.1. Model of Structural Signal

Multiple reflections in the laminate brings about severely overlapping and reverberant signals. Modelling the propagation path in the laminate is partly complicated. In the literature [17], a parametric layer model was proposed and applied in parameters estimation successfully. Here, the parametric layer model was introduced for structural signal modelling of CFRP laminate.

**Figure 1:**
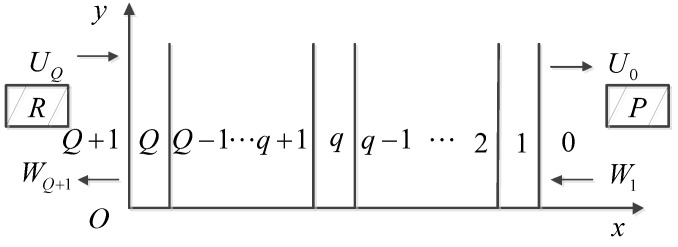
Model of multiple reflections.

The scheme of the parametric layer model is depicted in Figure 1. The multi-layered media consists of Q+2 layers, namely, layer 0~Q+1. Two transducers P and R are placed in medium 0 and Q+1. $W_{1}$ and $U_{Q}$ are corresponding transmitted signals and $U_{0}$ and $W_{Q+1}$ are received signals of P and R, respectively. In the pulse–echo model, only one transducer exists; here we set it as P, thus, $U_{Q}=0$. Apparently, $U_{0}(f)$ becomes the frequency response function of the laminate if we set the pressure amplitude of $W_{1}$ as 1.

The parametric layer model can be expressed using a system of linear equations in a block matrix as:

(1) $$\left[\begin{array}{c} W_{Q+1}\\ U_{Q-1}\\ \vdots \\ W_{q+1}\\ U_{q-1}\\ \vdots \\ W_{2}\\ U_{0} \end{array}\right]=\left[\begin{array}{cccccccc} A_{Q} & B_{Q} & & & \cdots & & & 0\\ C_{Q} & D_{Q} & & & & & & \\ & & \ddots & & & & & \\ & & & A_{q} & B_{q} & & & \vdots \\ \vdots & & & C_{q} & D_{q} & & & \\ & & & & & \ddots & & \\ & & & & & & A_{1} & B_{1}\\ 0 & & & \cdots & & & C_{1} & D_{1} \end{array}\right]\left[\begin{array}{c} U_{Q}\\ W_{Q}\\ \vdots \\ U_{q}\\ W_{q}\\ \vdots \\ U_{1}\\ W_{1} \end{array}\right],$$

where $A_{q}$, $B_{q}$, $C_{q}$, $D_{q}$ are:

(2) $$\begin{array}{c} A_{q}=\frac{(1-R_{q+1,q}^{2})R_{q,q-1}M_{q}{}^{2}}{1+R_{q,q-1}R_{q+1,q}M_{q}{}^{2}}\\ B_{q}=\frac{(1-R_{q+1,q})M_{q}}{1+R_{q,q-1}R_{q+1,q}M_{q}{}^{2}}\\ C_{q}=\frac{(1+R_{q+1,q})M_{q}}{1+R_{q,q-1}R_{q+1,q}M_{q}{}^{2}}\\ D_{q}=\frac{-R_{q+1,q}M_{q}{}^{2}}{1+R_{q,q-1}R_{q+1,q}M_{q}{}^{2}} \end{array},$$

where $R_{ij}$ is the reflection coefficient when the ultrasonic wave propagates from medium i into medium j. Suppose that the density and *P*-wave velocity are ρ and c, respectively, therefore:

(3) $$R_{ij}=\frac{Z_{j}-Z_{i}}{Z_{j}+Z_{i}}=\frac{\rho_{j}c_{j}-\rho_{i}c_{i}}{\rho_{j}c_{j}+\rho_{i}c_{i}},$$

$M_{q}$ is the model for the material properties inside the layer q, defined as:

(4) $$M_{q}=e^{-2d_{q}(i2\pi f/c_{q}+\alpha_{q}f^{2})},$$

where $\alpha_{q}$ is attenuation in layer q and $d_{q}$ is thickness of layer q. The factor $f^{2}$ denotes that the attenuation coefficient is proportional to the square of frequency in classical absorption theory.

### 2.2. Model of Scattering Signal

Scattering in CFRP laminate is usually complicated. To simplify the modelling, a set of assumptions are adopted: (1) multiple scattering does not happen, and the scattering of each void is independent; (2) the matrices of the laminate are all composed of carbon-fiber to get rid of the multiple reflections of scattered signal. Consequently, the scattering of each void is simplified to the case that scattering in an infinite-elastic solid medium, and the total scattering signal is superposition of scattering signal excited by each void.

**Figure 2:**
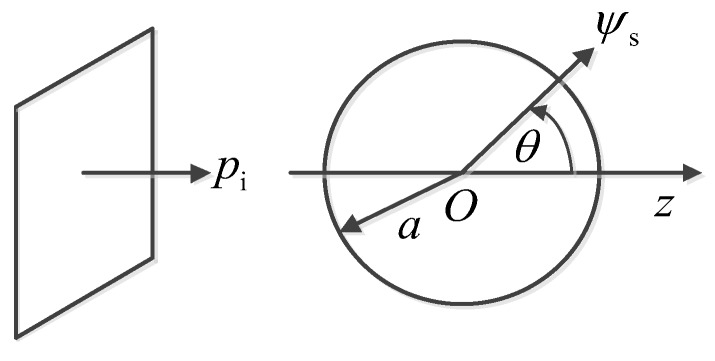
Scattering model of a void in elastic solid.

The scattering model depicted in Figure 2 illustrates the case where an incident wave $p_{i}$ is scattered by a spherical void (medium 2) with radius a. Suppose that the velocity of *P*-wave and *S*-wave in the matrix (medium 1) are $c_{1L}$ and $c_{1S}$, respectively, then:

(5) $$\begin{array}{cc} k_{1}=2\pi f/c_{1L}, & \kappa_{1}=2\pi f/c_{1S} \end{array},$$

where $k_{1}$ and $\kappa_{1}$ are the wavenumbers of *P*-wave and *S*-wave in medium 1, respectively.

More simplifications are introduced: (1) the radii of voids are so small that $k_{1}a\ll 1,\kappa_{1}a\ll 1$; (2) no wave mode exists inside the void; (3) only the scattering *P*-wave along π direction can be received by the transducer; and (4) the attenuation of the scattering signal are negligible. Obviously, this scattering model is rough and preliminary; however, it still works for us to investigate the characteristics of the scattering signal. 

For the normalized incident wave $p_{i}=exp(ik_{1}r\cos\theta )$, the scattering wave in the matrix can be expressed as:

(6) $$\psi_{s}=\sum_{m=0}^{\infty }A_{m}h_{m}{}^{\left(2\right)}(k_{1}r)P_{m}(\cos\theta ),$$

where $h_{m}{}^{\left(2\right)}(z)$ is the spherical Hankel function of the second kind, and $P_{m}(z)$ is the Legendre polynomials. For further simplification, in Equation (3), there exists θ=π, thus $P_{m}(\cos\theta )={\left(-1\right)}^{m}$.

The scattering coefficients $A_{m}$, after correcting the minus sign of $A_{1}$, can be expressed as [18]:

(7) $$\begin{array}{l} A_{0}=\frac{1}{3}[1-\frac{3}{4}{\left(\frac{\kappa_{1}}{k_{1}}\right)}^{2}]\frac{1}{k_{1}}{\left(k_{1}a\right)}^{3}\\ A_{1}=i\frac{1}{3}\frac{1}{k_{1}}{\left(k_{1}a\right)}^{3}\\ \cdots \\ A_{m}={\left(-i\right)}^{m}(4m^{2}-1){\left[2^{m}\frac{m!}{(2m)!}\right]}^{2}\frac{1}{1-\frac{2m^{2}+1}{2m(m-1)}{\left(\frac{\kappa_{1}}{k_{1}}\right)}^{2}}\frac{1}{k_{1}}{\left(k_{1}a\right)}^{2m-1} \end{array}$$

Allowing for the time-of-flight, the scattering frequency response function $V_{0}(f)$ can be expressed as:

(8) $$V_{0}(f)=\sum_{k=1}^{N_{v}}\left|\psi_{s}\right|\exp(-i2\pi f\frac{2r}{c_{1L}}),$$

where $N_{v}$ is the number of voids. The operator “|z|” denotes the module of complex z.

### 2.3. Model of Non-Acoustic Noise

The non-acoustic noise n(t) can usually be simply modeled as zero-mean white noise with Gaussian amplitude distribution. So, the power spectral density is equal to its variance $D_{n}$.

### 2.4. Model of Backscattered Signal

Suppose the spectrum of the transducer is $H_{t}(f)$, then the structural signal u(t) and scattering signal v(t) can be expressed as:

(9) $$\begin{array}{l} u(t)=\int_{-\infty }^{+\infty }H_{t}{}^{2}(f)U_{0}(f)e^{i2\pi ft}df\\ v(t)=\int_{-\infty }^{+\infty }H_{t}{}^{2}(f)V_{0}(f)e^{i2\pi ft}df \end{array}.$$

Subsequently, u(t) and v(t) are normalized as follows:

(10) z=z/max(|z|),

where z is a complex matrix.

For brevity, all “normalization” discussed in later sections are performed in the form of Equation (10) without specific instruction.

The backscattered signal x(t) of CFRP laminate can be expressed as:

(11) $$x\left(t\right)=n(t)+A_{u}u(t)+A_{v}v(t),$$

where $A_{u}$ and $A_{v}$ are amplitude factors.

## 3. VMD and GST 

### 3.1. VMD

The variational mode decomposition method decompose the input signal into a determined number of modes. Each mode is compact around a center pulsation. The modes and center frequencies are extracted by solving the constrained variational problem as follows:

(12) $$\begin{array}{r} \underset{\left\{u_{k}\},\{\omega_{k}\right\}}{\min}\left\{\sum_{k}{\left\|\partial_{t}\left[\left(\delta (t)+\frac{i}{\pi t}\right)*u_{k}(t)\right]e^{-i\omega_{k}t}\right\|}_{2}^{2}\right\}\\ s.t.\;\sum_{k}u_{k}=f \end{array},$$

where $\left\{u_{k}\}=\{u_{1},u_{2},\ldots ,u_{K}\right\}$ and $\left\{\omega_{k}\}=\{\omega_{1},\omega_{2},\ldots ,\omega_{K}\right\}$ are the set of all modes extracted and their center frequencies, respectively. f is the input signal. δ is the Dirac distribution and * denotes convolution.

Using quadratic penalty term α and Lagrangian multipliers λ(t), the above constrained optimization problem can be changed to an unconstrained optimization problem. The changed formulation with the augmented Lagrangian L can be expressed as follows:

(13) $$\begin{array}{l} L\left(\left\{u_{k}\right\},\left\{\omega_{k}\right\},\lambda \right)=\alpha \sum_{k}{\left\|\partial_{t}\left[\left(\delta (t)+\frac{i}{\pi t}\right)*u_{k}(t)\right]e^{-i\omega_{k}t}\right\|}_{2}^{2}+\\ {\left\|f(t)-\sum_{k}u_{k}(t)\right\|}_{2}^{2}+\langle \lambda (t),f(t)-\sum_{k}u_{k}(t)\rangle \end{array}$$

The saddle point of Equation (13) can be solved by alternate direction method of multipliers. This means that the optimal solution of Equation (12) can be gained. The completed steps of VMD are given as follows.

- Step 1: Initialize $\left\{u_{k}^{1}\right\},\left\{\omega_{k}^{1}\right\},\lambda^{1},n\leftarrow 0$.
- Step 2: n←n+1.
- Step 3: Update ${\hat{u}}_{k}$ and $\omega_{k}$ by iterating through k=1,2,⋯,K.
  (14) $${\hat{u}}_{k}^{n+1}(\omega )\leftarrow \frac{\hat{f}(\omega )-\sum_{i<k}{\hat{u}}_{i}^{n+1}(\omega )-\sum_{i<k}{\hat{u}}_{i}^{n}(\omega )+\frac{1}{2}{\hat{\lambda }}^{n}(\omega )}{1+2\alpha {\left(\omega -\omega_{k}^{n}\right)}^{2}},$$
  (15) $$\omega_{k}^{n+1}\leftarrow \frac{\int_{0}^{\infty }\omega {\left|{\hat{u}}_{k}^{n+1}(\omega )\right|}^{2}d\omega }{\int_{0}^{\infty }{\left|{\hat{u}}_{k}^{n+1}(\omega )\right|}^{2}d\omega }.$$
- Step 4: Update λ.
  (16) $${\hat{\lambda }}^{n+1}(\omega )\leftarrow {\hat{\lambda }}^{n}(\omega )+\tau \left(\hat{f}(\omega )-\sum_{k}{\hat{u}}_{k}^{n+1}(\omega )\right).$$
- Step 5: For the predefined tolerance ε>0, repeat from Step 2 to Step 4 until convergence.
  (17) $${{\sum_{k}\left\|{\hat{u}}_{k}^{n+1}-{\hat{u}}_{k}^{n}\right\|}_{2}^{2}/\left\|{\hat{u}}_{k}^{n}\right\|}_{2}^{2}<\varepsilon .$$

### 3.2. GST

The S-transform (ST) derived by Stockwell et al. [15] demonstrates the great superiority of multi-resolution analysis in respect to short-time Fourier transform and the advantages of phase-preserving property compared with wavelet transform. However, in some cases, ST may output poor results due to the fixed shape of the Gaussian window. Generalized Stockwell transform is the inheritance and development of ST by introducing extra parameters in ST for window-shape adjustment. Here, we adopt the definition in the literature [16], as shown below:

(18) $$S(\tau ,f;a_{s},b_{s})=\int_{-\infty }^{+\infty }x(t)\frac{\left|f\right|}{(a_{s}f+b_{s})\sqrt{2\pi }}e^{\frac{-{\left(\tau -t\right)}^{2}f^{2}}{2{\left(a_{s}f+b_{s}\right)}^{2}}}e^{-i2\pi ft}dt,$$

where $a_{s}$ is the slope and $b_{s}$ is the intercept.

## 4. Numeric Simulation

### 4.1. Parameters Setting in the Simulation

**Figure 3:**
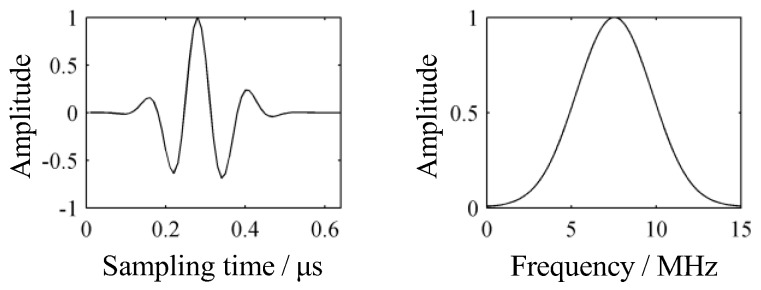
Transmission pulse signal and spectrum of transducer.

The center frequency of the transducer used in the simulation was 7.5 MHz. The transmission pulse signal and normalized spectrum of the transducer are shown in Figure 3.

The material parameters adopted are listed in Table 1. Some of them came from experimental results obtained by Martin [1], and others were set empirically. $\rho_{f}$, $c_{\mathrm{fL}}$, $c_{\mathrm{fS}}$, and $d_{f}$ are nominal values of density, *P*-wave velocity, *S*-wave velocity, and thickness of the fiber layer, respectively; $\rho_{r}$, $c_{r}$, and $d_{r}$ are nominal values of density, *P*-wave velocity, and thickness of the resin layer respectively. $N_{f}$ is number of plies. δ is the maximum deviation of a single ply thickness. The actual plies of thickness are set to normally distributed on the interval $d_{0}\cdot [1-\delta ,1+\delta ]$, where $d_{0}$ is $d_{f}$ or $d_{r}$.

Now the parameters adopted for the simulation of porosity and rich-resin are introduced.

First, the concept “linear density” denoted by ρ is introduced for localized porosity description:

(19) $$\rho =\left[\frac{N_{v0}}{d_{2}-d_{1}}\right],$$

where $\left[d_{1},d_{2}\right]$ is the interval where the voids exist and $N_{v0}$ is number of voids. The operator “[]” means rounding.

Assume that the intervals between the transducer and CFRP laminate is $\left[d_{\min},d_{\max}\right]$ and $d_{\min}$ has been predefined, then:

(20) $$d_{\max}=d_{\min}+\sum_{i=1}^{N_{f}}d_{fi}+\sum_{i=1}^{1+N_{f}}d_{ri}.$$

Ideally, all voids are located in $\left[d_{\min},d_{\max}\right]$. However, this will cause a sharp burst at $r=d_{\min}$ in the scattering signal. To avoid this, here the interval $\left[d_{\min},d_{v1}\right]$ is expanded to $\left[d_{\min}^{*},d_{v1}\right]$, where $d_{\min}^{*}=d_{\min}/2$. So, for the uniformly distributed voids (homogenous porosity), the linear density is:

(21) $$\rho_{0}=\left[\frac{N_{v}}{d_{\max}-d_{\min}^{*}}\right].$$

Here, we assumed that the localized porosity was located at layers $n_{v1}\sim n_{v2}$. Similarly, the related intervals $\left[d_{v1},d_{v2}\right]$ could be obtained, which was used for truncation of scattering signals.

The linear density of localized porosity is defined as $\rho_{1}=\lambda_{v}\rho_{0}$, where $\lambda_{v}(\lambda_{v}>1)$ is the factor. So the number of voids in $\left[d_{v1},d_{v2}\right]$ is $\lambda_{v}\rho_{0}(d_{v2}-d_{v1})$. The linear density in $\left[d_{\min}^{*},d_{v1}\right]$ and $\left[d_{v2},d_{\max}\right]$ is

(22) $$\rho_{2}=\left[\frac{N_{v}-\lambda_{v}\rho_{0}(d_{v2}-d_{v1})}{\left(d_{v1}-d_{\min}^{*})+(d_{\max}-d_{v2}\right)}\right].$$

The distances between the transducer and the voids were set to be uniformly distributed in each interval.

Similarly, for the rich-resin, only one ply of rich-resin was considered, and a factor $\lambda_{r}(\lambda_{r}>1)$ was set, which meant that the thickness of the ply with rich-resin was $\lambda_{r}d_{r}$; the ply was located at layer $n_{r}$.

The porosity and rich-resin parameters adopted are listed in Table 2.

### 4.2. Simulation of Typical Cases

Four typical cases were investigated in which the CFRP laminate had: (1) no defect; (2) rich-resin; (3) homogenous porosity; and (4) localized porosity. To eliminate the effects of non-acoustic noise, here n(t) was omitted.

**Figure 4:**
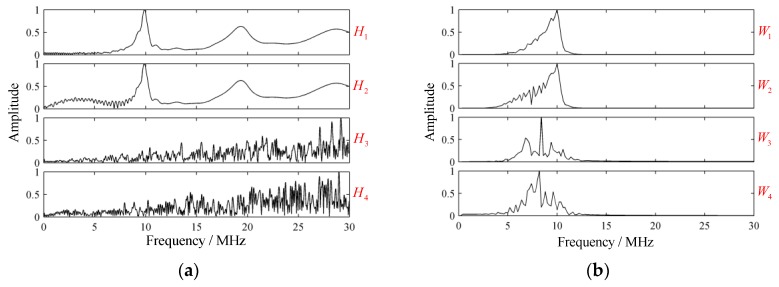

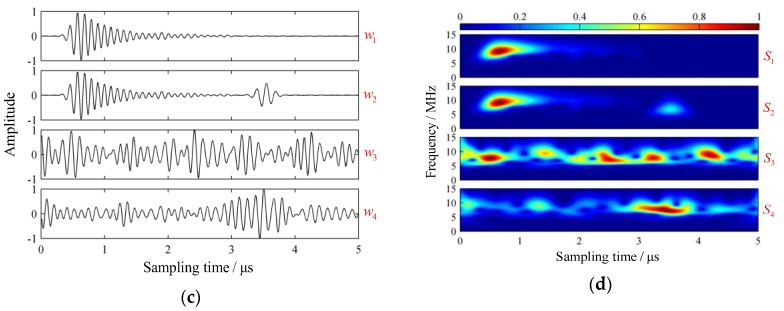
Simulated results of four typical cases. (**a**) Frequency response functions; (**b**) frequency spectrums; (**c**) waveforms; and (**d**) generalized Stockwell transform spectrums.

For the cases (1–4), the frequency response function $H_{i}(f)$ and frequency spectrum $W_{i}(f)$, waveform $w_{i}(t)$, GST spectrum $S_{i}(\tau ,f)$ under excitation of $h_{t}(t)$ could be obtained, where i=1,2,3,4. Simulated results are visualized in Figure 4, in which $H_{i}$, $W_{i}$, $w_{i}$, and $S_{i}$ were normalized.

For case (1), the resonance occurred due to the interferences of multiple reflection echoes. The resonance frequency was around 10 MHz. It was reported [13] that, for layered structures, the resonance frequencies $f_{m}$ were:

(23) $$\begin{array}{cc} f_{m}\approx \frac{mc_{\mathrm{fL}}}{2d_{f}}, & m=1,2,\ldots \end{array}.$$

**Figure 5:**
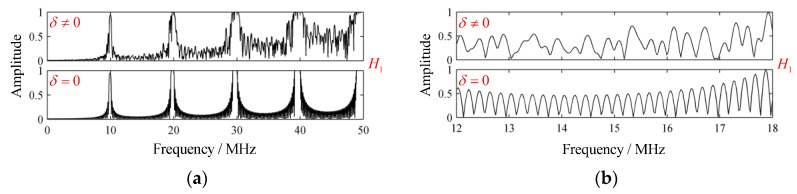
The frequency response function $H_{1}$ without attenuation. (**a**) $H_{1}$ and (**b**) local enlarged drawings of $H_{1}$.

So there existed $f_{1}\approx 10$ MHz, which coincided with the simulated structural signal well. A sufficient bandwidth was essential for excitation of strong resonance signal. Due to the attenuation in the laminate, the structural signal declined and disappeared. The $H_{1}$ with no attenuation occurring is presented in Figure 5a, where periodic spectral peaks can be observed, showing accordance with Equation (23). As a supplementary example, the special case, where the variations in ply thickness were omitted, was presented as well. It can be seen that the variations in ply thickness strongly increased the complexity of $H_{1}$, whose local enlarged drawings within the frequency intervals 12~18 MHz are presented in Figure 5b for clarity.

For cases (2–4), the existence of porosity and rich-resin both generated echoes in the backscattered signal. The center frequencies of echoes were around or below that of the transducer. The center frequencies often showed a declining trend for the frequency-dependent attenuation. The localized porosity and rich-resin only generated few apparent echoes in their locations; the homogenous porosity generated multiple low-amplitude echoes throughout the backscattered signal, leading to heavy overlapping and reverberation. 

Due to the fact that the resonance frequency is usually higher than the center frequency of the transducer, it can be inferred that the center frequency of the backscattered signal will decrease when the porosity or rich-resin increases. This inference has been verified in the literature [8].

Based on the analysis of flaw echoes whose center frequencies lie around that of the transducer in the backscattered signal, localized defects in laminate may be revealed. However, detecting those flaw echoes is often challenging since they are not as clear as echoes caused by delamination or holes.

Another challenge is distinguishing between localized porosity and rich-resin. This becomes a tough task as the echoes excited by them are similar both in the time domain and the frequency domain. As most researchers do, here we do not work on this kind of distinguishing task. 

Limitations of the backscattered signal model are explicit. The neglect of non-linear behaviors, the simplifications in scattering signal modelling, etc., may lead to certain differences between simulated signals and experimental signals, and of course, the backscattered signal model is unsuited to quantitative analysis of defects. Even so, the backscattered signal model is capable of revealing the time-frequency properties of the backscattered signal, which is conducive to defects detection in CFRP laminate.

### 4.3. Model Simplification

According to the analysis above, two conclusions can be made: (1) serious localized porosity and rich-resin would generate flaw echoes whose center frequencies lied around that of transducer; and (2) the structural signal often tended to be damped oscillatory with center frequency around $f_{1}$.

Any echo s(θ;t) can be expressed by the asymmetric Gaussian chirplet model [19] as follows:

(24) $$\begin{array}{c} s(\theta ;t)=a(t-\tau )\cos(\phi (t-\tau ))\\ a(t)=\exp(-\alpha (1-r\tanh(\kappa t))t^{2})\\ \phi (t)=2\pi f_{c}t+\psi t^{2}+\varphi \end{array},$$

where $\theta =[\alpha ,r,\kappa ,\tau ,f_{c},\psi ,\varphi ]$ is the parameter vector, α is bandwidth factor, r is asymmetric factor, tanh(κt) is the hyperbolic tangent function of order κ, τ is time of flight, $f_{c}$ is center frequency, ψ is chirplet factor, and φ is initial phase. To avoid confusion, θ and ϑ are used to denote parameter vectors in flaw echoes and structural signal, respectively.

The spectrum of scattering signal arising from homogenous porosity is similar to that of grain scattering signal in metallic materials. In the Rayleigh region, the frequency spectrum of backscattered signal can be expressed as [20]:

(25) $$\begin{array}{l} V_{0}(f)=\sum_{k=1}^{N_{v}}\beta_{k}\frac{{\left(2\pi f\right)}^{2}}{x_{k}}e^{-\alpha_{s}2x_{k}{\left(2\pi f\right)}^{4}}e^{-i(2\pi f)\frac{2x_{k}}{c_{\mathrm{lL}}}}\\ v(t)=\int_{-\infty }^{+\infty }H_{t}{}^{2}(f)V_{0}(f)e^{i2\pi ft}df \end{array}$$

where $\alpha_{s}$ is the material attenuation coefficient, $\beta_{k}$ is scattering coefficients of the $k_{\mathrm{th}}$ scatterer, which are generally set to be normally distributed, $x_{k}$ is the position of the $k_{\mathrm{th}}$ scatterer and is set to be uniformly distributed, v(t) is subsequently normalized.

Based on the above analysis, the simplified backscattered signal model can be expressed as:

(26) $$\begin{array}{l} x\left(t\right)=n(t)+A_{u}u(\vartheta ,t)+A_{v}v(t)+s(t)\\ s(t)=\sum_{i=1}^{N}A_{si}s_{i}(\theta_{i},t) \end{array},$$

where $A_{si}$ are the amplitude factors and N is number of flaw echoes.

**Figure 6:**
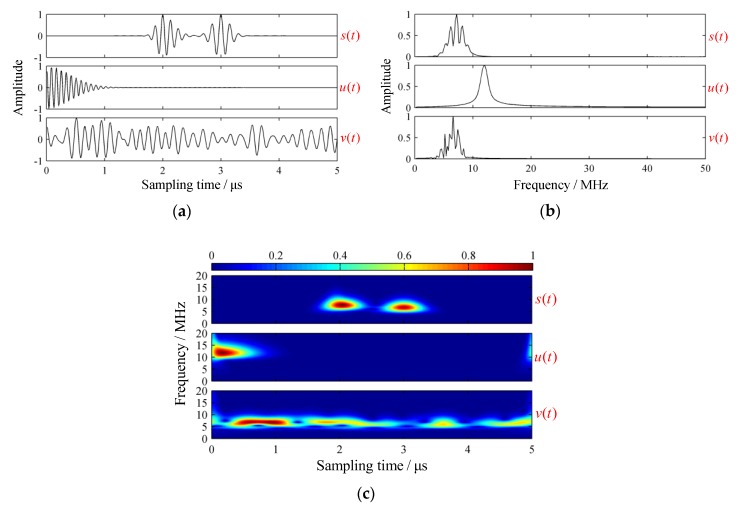
Simulated results of a simplified backscattered signal model. (**a**) Waveforms; (**b**) spectrums; and (**c**) GST spectrums.

As an instance, the typical results of normalized waveforms, spectrums, and GST spectrums are visualized in Figure 6.

Clearly, the simplified model could reveal time-frequency properties of backscattered signals to a certain degree. However, tuning parameters appropriate for simulation might be confusing and time consuming.

## 5. Experimental Study

### 5.1. Experimental System

**Figure 7:**
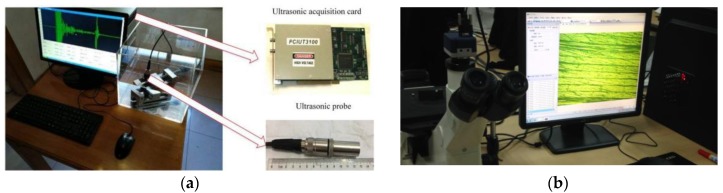
Experimental system. (**a**) Ultrasonic test system; (**b**) metallographic microscope system.

The ultrasonic test system and the metallographic microscope system are shown in Figure 7. The sampling frequency was 50 MHz. The experimental signals were acquired from CFRP laminates provided by an aircraft manufacturing company. The number of plies of the specimen was $N_{f}=72$. The thickness of the fiber ply and resin layer were 0.125 mm and 0.005 mm, respectively. Metallographic observation of other CFRP specimens in the same batch showed that no obvious porosity was observed in the CFRP sample, and the porosity was close to zero, but rich-resin defects were observed in a few local locations.

### 5.2. Analysis of Backscattered Signals

**Figure 8:**
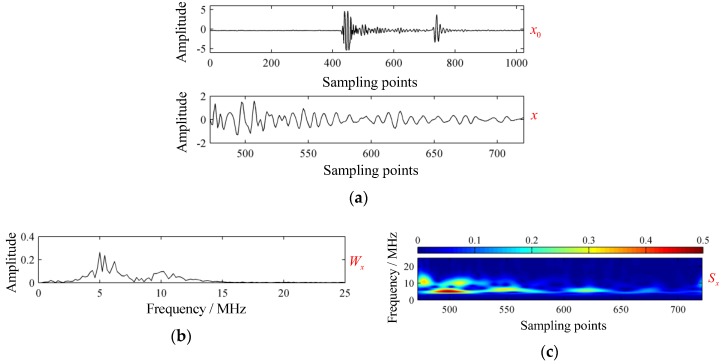
Acoustic characterization of experimental signal. (**a**) Waveform; (**b**) spectrum; and (**c**) GST spectrum.

The acquired signal $x_{0}(t)$, backscattered signal x(t), corresponding spectrum $W_{x}$, and GST spectrum $S_{x}$ are shown in Figure 8. The time of flight Δt could be estimated by:

(27) $$\Delta t\approx \frac{M_{1}-M_{0}}{2f_{s}}\approx \frac{N_{f}d_{f}}{c_{\mathrm{fL}}}.$$

According to Equation (23), the resonance frequency $f_{1}$ could also be estimated by:

(28) $$f_{1}\approx \frac{N_{f}}{M_{1}-M_{0}}f_{s}.$$

where $M_{0}$ and $M_{1}$ were the central sampling points of the front-wall echo and back-wall echo. Here, we did not use the sampling points where the ultrasonic wave first arrived at the front-wall and back-wall to keep consistency with the defect localization method described later. In Figure 8a, $M_{0}\approx 449$ and $M_{1}\approx 740$ could be obtained, thus $f_{1}\approx 12$ MHz.

The structural signal with resonance frequency around 10–12 MHz and a flaw echo with center frequency around 5–6 MHz could be detected clearly in Figure 8c. To investigate the modes individually, we used VMD for modes extraction.

**Figure 9:**
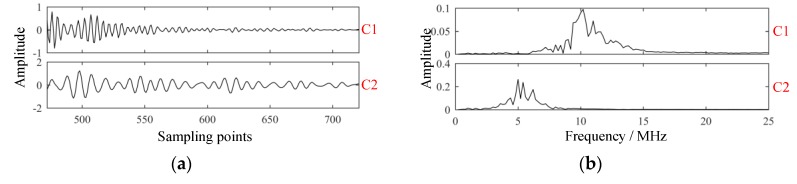

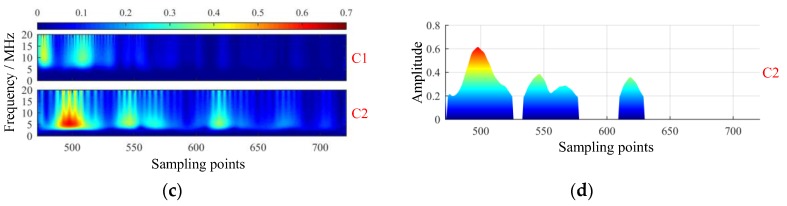
Modes extracted of the experimental signal via variational mode decomposition. (**a**) Intrinsic mode functions; (**b**) spectrums of intrinsic mode functions; (**c**) GST spectrums of intrinsic mode functions; (**d**) projection of GST spectrum of C2.

**Figure 10:**
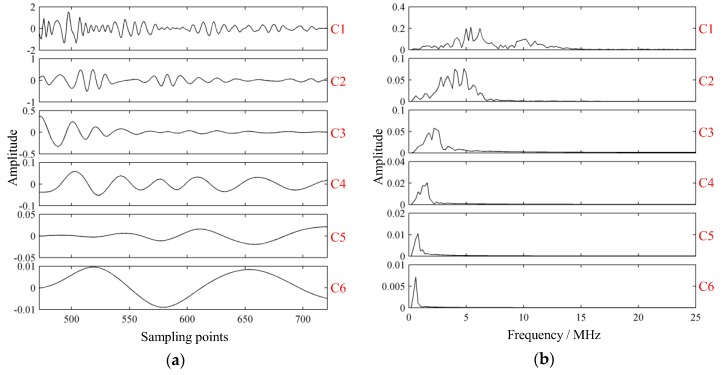
Modes extracted of the experimental signal via empirical mode decomposition. (**a**) Intrinsic mode functions; (**b**) spectrums of intrinsic mode functions.

Modes extracted by VMD with parameters K=2 and α=64 are shown in Figure 9. Comparably, the modes extracted by empirical mode decomposition (EMD) [21] are shown in Figure 10.

Clearly, results obtained via EMD suffered mode mixing in C1 and some false modes could also be detected. As shown in Figure 10b, EMD was a screening process from high to low, with the frequency of each component decreasing successively, and the frequency of C2–C6 approximately satisfied the binary filtering characteristic. In the process of EMD, mode mixing occurred, and some components of C2 were decomposed to C1. In this way, C1 became a multi-component signal, whose main components included resonant structure noise and components close to the probe frequency, while C2 was damaged. The occurrence of mode mixing meant that the decomposition results of EMD were not ideal.

Conversely, modes were extracted by VMD successfully. As shown in Figure 9, C1 was the structural signal, and C2 was made up of scattering signal and flaw echoes. In Figure 9c, four local defects were revealed from the GST spectrums. To decide the locations of defects, a threshold operation was first performed on the GST spectrum, and projection of GST spectrums onto the time-amplitude plane was implemented subsequently, as shown in Figure 9d. From Figure 9d, the sampling points m of those four defects were 497, 546, 567, and 619, respectively. Correspondingly, defect plies n could be estimated by:

(29) $$n\approx \frac{m-M_{0}}{M_{1}-M_{0}}N_{f}.$$

**Figure 11:**
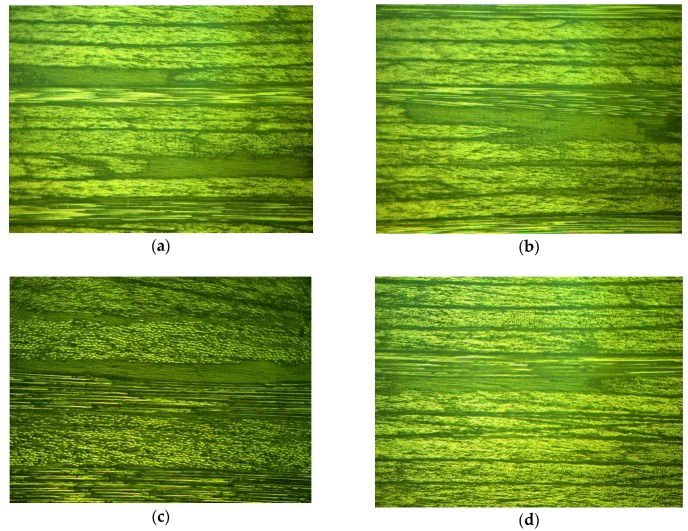
Rich-resin areas in the specimen. (**a**) Layer: 10 and 14; (**b**) layer: 25; (**c**) layer: 31; and (**d**) layer: 42.

Yielding n≈12,24,29,42, respectively. Metallographic experiment was conducted to check the localized defects in the laminate. Five obvious areas of rich-resin were founded at plies $n_{0}=10,14,25,31,42$. The photomicrographs of the specimen are shown in Figure 11, showing certain coincidence with the mode C2. The first two defective plies, i.e., plies 10 and 14, had not been separated: they were close to each other and near the front-wall surface. Interestingly, serious porosity was hardly found in the specimen, although many researchers reported that porosity was far more common than rich-resin in CFRP laminate.

It should be noted that appropriate parameters K and α were required for modes extraction. Currently, K and α were both selected by trial and error. Seeking for automatic and intelligent methods for determination of K and α in CFRP backscattered signal processing is a tough task and is still under way. In fact, selecting K and α manually is often not so difficult based on the backscattered signal model.

**Figure 12:**
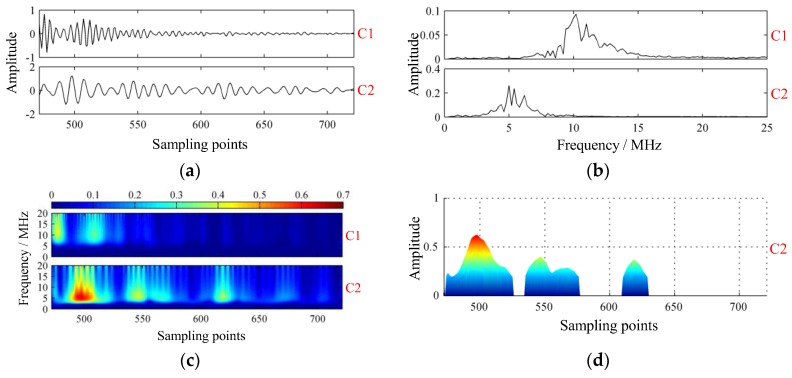
Modes extracted by VMD with parameters K=2 and α=4. (**a**) Intrinsic mode functions; (**b**) spectrums of intrinsic mode functions; (**c**) GST spectrums of intrinsic mode functions; and (**d**) projection of GST spectrum of C2.

**Figure 13:**
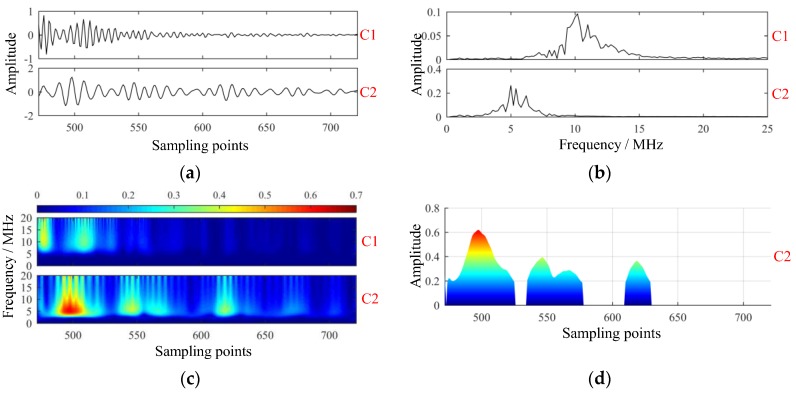
Modes extracted by VMD with parameters K=2 and α=16. (**a**) Intrinsic mode functions; (**b**) spectrums of intrinsic mode functions; (**c**) GST spectrums of intrinsic mode functions; and (**d**) projection of GST spectrum of C2.

The K and α selected in this paper were suboptimal. There is no strict “optimal value” in fact. The reason why K=2 was selected was that the frequency components of the collected ultrasonic detection signals were mainly composed of: (1) the components close to the probe frequency, mainly composed of direct reflection signals and scattering signals; and (2) resonance structure noise and other high-frequency components. Experiments showed that when K=2, the value range of α was relatively wide, that was, $\alpha =2^{j}(1\leq j\leq 12)$. When α=4 and α=16, the results of VMD decomposition are shown in Figure 12 and Figure 13, respectively.

**Figure 14:**
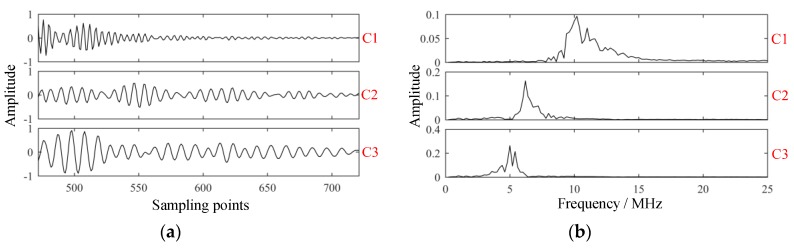

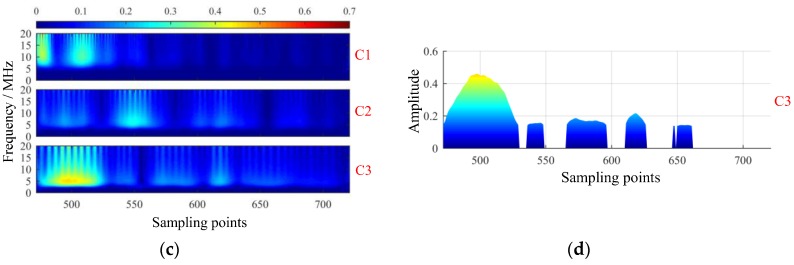
Modes extracted by VMD with parameters K=3 and α=16. (**a**) Intrinsic mode functions; (**b**) spectrums of intrinsic mode functions; (**c**) GST spectrums of intrinsic mode functions; and (**d**) projection of GST spectrum of C2.

When K=3, mode splitting occurs. For example, when α=16, the decomposition results are shown in Figure 14. Obviously, C1 was still mainly composed of high-frequency components such as resonant structure noise, while C2 and C3 were actually components close to the probe frequency. Mode splitting occurred. At this time, the decomposition results were not ideal and should not be applied to detection.

## 6. Conclusions

The modelling of backscattered signal in CFRP laminate was investigated. The parametric layer model was introduced for structural signal modelling. It showed that if there was sufficient bandwidth, resonance would occur in the laminate due to the interferences of multiple reflection echoes and the rich-resin tended to generate flaw echo where it located.

The scattering signal model was established based on superposition of the scattering signal excited by each void. It was shown that the localized porosities generated few apparent echoes where they located, while the homogenous porosity generated multiple low-amplitude echoes throughout the backscattered signal. Since center frequency of echoes were usually lower than resonance frequency, the center frequency of backscattered signal would decrease when the porosity increased.

A simplified backscattered signal model was then put forward, showing some potential in revealing time-frequency properties of backscattered signal. The VMD method was used for defect mode extraction, successfully avoiding the mode mixing and false modes easily existing in EMD. Then, the GST method was adopted for localizing the defects, showing sufficient flexibility.

## Figures and Tables

**Table 1:** Parameters of materials.

$\rho_{f}(kg/m^{3})$	$c_{\mathrm{fL}}(m/s)$	$c_{\mathrm{fS}}(m/s)$	$d_{f}(mm)$	$\rho_{r}(kg/m^{3})$	$c_{r}(m/s)$	$d_{r}(mm)$	$N_{f}$	δ
1690	3077	1770	0.150	1270	2903	0.005	48	5%

**Table 2:** Parameters of porosity and rich-resin.

*a* (μm)	*N*_v_	*d*_min_ (cm)	*n*_v1_	*n*_v2_	*λ*_v_	*n*_r_	*λ*_r_
10	3000	1.0	29	31	5	30	8
